# Waveform analysis of differential graphs of reconstructed impedance cardiography from healthy individuals

**DOI:** 10.1111/anec.12714

**Published:** 2019-11-01

**Authors:** Bai‐qing He, Zi‐ming Wang, Shi‐jiang Kuang, Qiu‐jin Xiao, Ming‐xing Kuang, Juan‐Feng Ji, Yun‐qiang Wu

**Affiliations:** ^1^ Department of Electronic Engineering Nanchang Institute of Technology Nanchang China; ^2^ Department of Special Diagnosis 908th Hospital Nanchang China

**Keywords:** differential graph, reconstructed impedance cardiography, time interval, wave amplitude

## Abstract

**Purpose:**

The aim is to measure and analyze the wave amplitudes and time intervals of differential graphs of reconstructed impedance cardiography (RICG).

**Methods:**

180 adults with normal cardiac function between the ages of 18–78 were included in the study. Six mingled impedance changes on chest surface were simultaneously detected for each subject. The differential graphs of five impedance change components of RICG were obtained through waveform separation and software differentiation. The amplitudes of C, X, O, b waves and time intervals of Q‐b and Q‐C were measured and statistically analyzed.

**Results:**

The amplitudes of C and X waves in PL, PR, AO, and that of C, O, b waves in LV and RV, all decrease as age increases. Wave amplitudes of the female group were bigger than those of the male group (*p* < .01), while the Q‐C intervals of the female group were shorter than that of the male group (*p* < .01). Among five impedance change components, the wave amplitude of AO was larger than those of PL and PR (*p* < .01), and wave amplitudes of PL and PR were bigger than those of LV and RV (*p* < .01). Q‐C intervals of LV and RV were longer than those of AO, PL and PR (*p* < .01), while the Q‐b intervals of LV and RV were shorter than the Q‐C intervals of AO, PL, and PR.

**Conclusions:**

The differential graphs of RICG could reflect indirectly the physiological activities and pathological changes of the heart and of the large blood vessels in thorax.

## 1 INTRODUCTION

Impedance cardiography (ICG), also known as the thoracic impedance graph, is an impedance change curve measured on the chest surface. It includes the affluent information concerning the physiological activity and pathological changes in the heart and of the large blood vessels in thorax. Now, the ICG is generally measured by means of Kubicek's method (Kubicek, Karnegis, Patterson, Witeose, & Mattson, 1966). The cardiac function information can be detected using the ICG, and hemodynamic changes in at‐risk patients also can be continuously monitored (D'Ambrosio et al., 2018; DeMarzo, 2013; Ebrahim et al., 2016; Heinink, Lund, & Williams, 2015; Rada, Cuffaro, & Galarza, 2014; Wang et al., 2017). Many studies have confirmed that the ICG measured using the above methods is a thoracic mingled impedance change, which mainly derives from volume changes in the aorta, blood vessels in lungs, and ventricles (Baker & Mistry, 1981; Patterson, 1985; Wang & Patterson, 1995). Obviously, the results measured with traditional ICG lack the specificity for cardiac functional assessment of left ventricle.

In order to solve this problem, we have derived five impedance change components corresponding to aorta (AO), left lung vasculature (PL), right lung vasculature (PR), left ventricle (LV), and right ventricle (RV) from six mingled impedance changes measured on the chest surface through waveform separation (Kuang et al., 2010; Ming‐Xing, Qiu‐Jin, Nan‐Zhen, Chao‐Ying, & Ai‐Rong, 2011). The waveform graphs of these components are collectively referred to as reconstructed impedance cardiography (RICG). The differential graphs of the RICG for 180 normal adults were measured in this paper, and the wave amplitudes and time intervals of the differential graphs of RICG were statistically analyzed.

## 2 METHODS

### 2.1 Measurement of thoracic mixed impedance change

#### 2.1.1 Placement method of impedance detected electrodes

**Figure 1:**
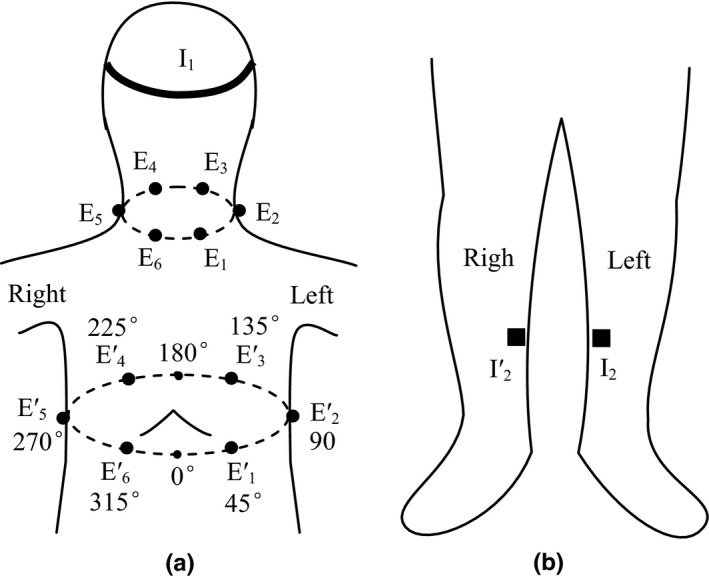
Placement method of impedance detected electrodes

The thoracic mixed impedance changes and basic impedances were measured using six leads consisting of 15 electrodes (Kuang, Xiao, He, Jing‐Juon, & Kuang, 2014; Ming‐Xing et al., 2011). The placement method of each electrode is shown in Figure 1. In which, I_1_, I_2_, and I_2_' are current electrodes dedicated to injecting a current. I_1_ is a semi‐ring strip silvered copper electrode which covers the subject's forehead, while I_2_ and I_2_' are silvered copper rectangle electrodes attached the left and right lower legs. 12 black circular dots indicate disposable Ag/AgCl voltage electrodes. E_1_, E_2_, E_3_, E_4_, E_5_, and E_6_ are the neck voltage electrodes applied to the neck root, and E_1_', E_2_', E_3_', E_4_', E_5_', and E_6_' are the chest voltage electrodes applied to the chest surface at xiphoid level.

#### 2.1.2 Basic composition of the measurement instrument

**Figure 2:**
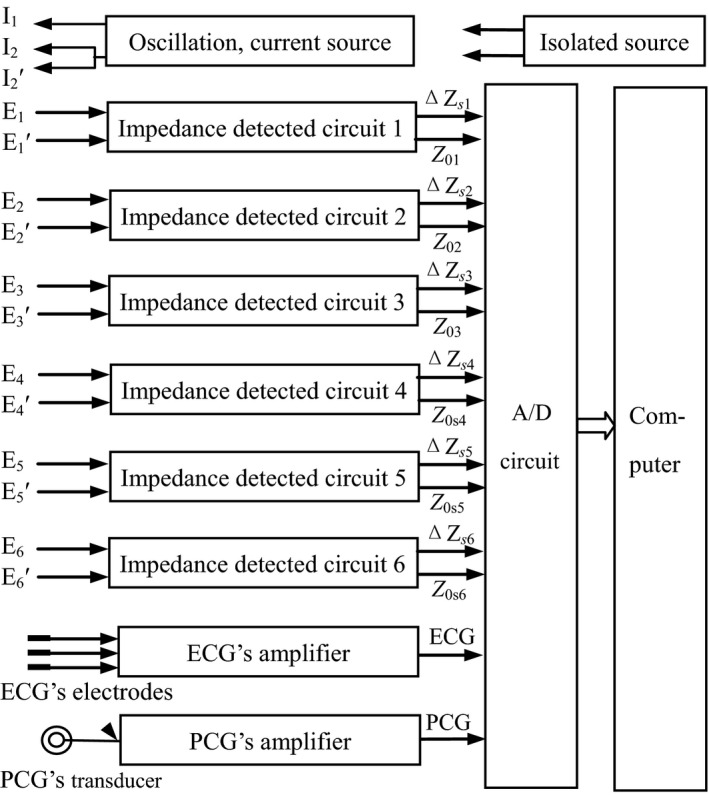
Basic composition of measurement instrument

Our research group developed the measurement instrument used in the present study. As shown in Figure 2, the hardware included a crystal oscillation circuit, an alternating current source circuit, six impedance detection circuits with same structure and properties, an amplifier for the electrocardiogram (ECG), an amplifier for the phonocardiogram (PCG), an A/D transform circuit, an isolated direct current source, a computer, an impedance lead wire, and an ECG lead wire. Operation of the support software consisted of the following: Input information regarding the human body, observe and record six thoracic mixed impedance changes and six basic impedances as well as the ECG and PCG, mark the starting point of ECG's Q wave and second heart sound (S_2_) for four consecutive cardiac cycles by artificial operation, correct the basic lines of thoracic mixed impedance changes, separate five impedance change components of AO, PL, PR, LV, and RV of RICG from six mingled impedance changes measured on chest surface through establishing and solving the thoracic impedance equations, make the differential treatment for each component, measure the waveform parameters of each differential graph, display, and print the waveform graphs and parameters.

#### 2.1.3 Measurement of RICG's differential graphs

Height, weight, chest circumference, blood pressure, as well as name, sex, age, and other relevant information were entered into the instrument prior to the measurement. The chest circumference was measured on the chest surface at the xiphoid level. A supine position was adopted for the measurement. The electrodes for sensing the ECG were attached to the right upper arm and left and right lower legs (i.e., Ⅱ lead mode). The transducer for sensing the PCG was placed at the cardiac apex on chest surface. The subject was asked to hold his/her breath for the duration of recording the waveform graphs. Thoracic mixed impedance changes (Δ*Z*_s1_, Δ*Z*_s2_, Δ*Z*_s3_, Δ*Z*_s4_, Δ*Z*_s5_, and Δ*Z*_s6_) and basal impedances (*Z*_0s1_, *Z*_0s2_, *Z*_0s3_, *Z*_0s4_, *Z*_0s5_, and *Z*_0s6_) of all six leads, as well as the ECG and PCG, were detected and displayed simultaneously.

**Figure 3:**
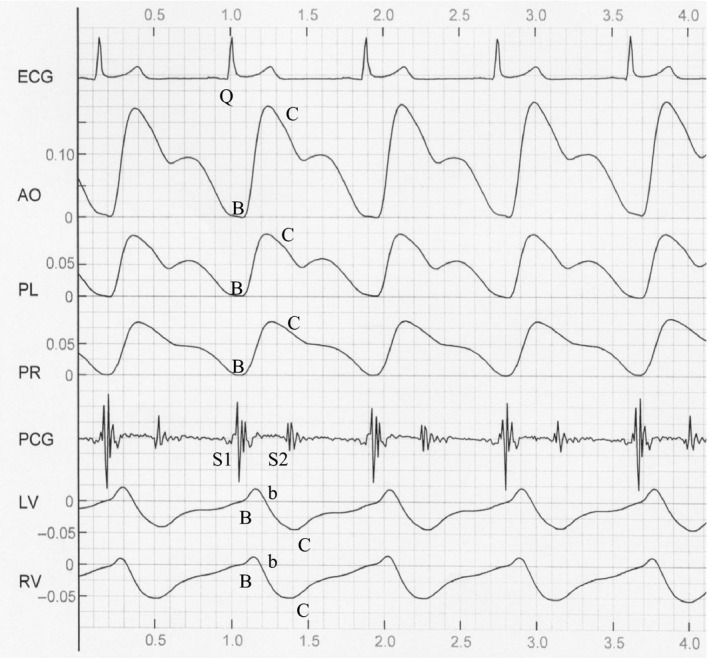
Waveform graphs of impedance change components of RICG

**Figure 4:**
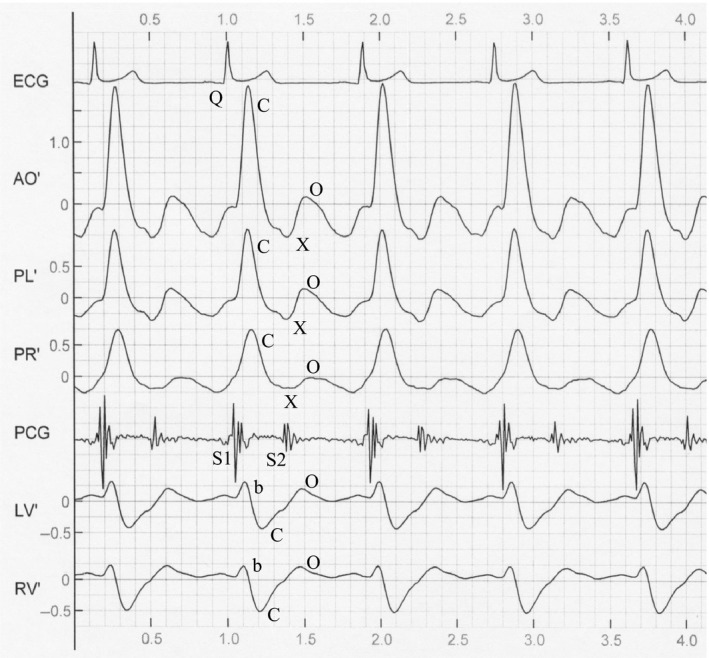
RICG's differential graphs

The starting point of the Q wave and S_2_ of the corresponding cycle were marked for four consecutive cardiac cycles in the waveform graphs by the artificial operation (Ming‐Xing et al., 2011). According to the mathematical methods in reference (Ming‐Xing et al., 2011), five impedance change components of AO, PL, PR, LV, and RV of RICG were separated from six mixed impedance changes measured on chest surface (see Figure 3). The differential graphs of each impedance change component of RICG could be further obtained through software differentiation (see Figure 4). The waveform graphs of impedance change components in Figure 3 were only a transition waveform in the measurement, and their differential graphs were usually applied in the measurement of cardiac function. Thus, only the waveform parameters of RICG's differential graphs were measured and analyzed in this paper.

As shown in Figure 4, lines 2, 3, and 4 are the differential graphs corresponding to the AO, PL, and PR components of RICG. They all have a high positive C wave in systolic phase, a negative X wave that appears after C wave, and a positive or negative O wave appears in diastolic prophase. Lines 6 and 7 are the differential graphs of LV and RV components, respectively. A small positive b wave appears in initial systolic stage; then, a larger negative C wave appears that is essentially in opposite phase of C wave of AO, PL, and PR. After the negative C wave, a positive O wave appears, and its amplitude is larger. There is no X wave in differential graphs of LV and RV. Lines 1 and 5 were the ECG and PCG, respectively, and they were used to measure the relative time intervals. As shown in Figure 4, the time interval from the initial point of the Q wave to C wave peak of various components is referred to as the Q‐C interval, and that from the initial point of ECG's Q wave to b wave peak of LV and RV is referred to as the Q‐b interval. The amplitudes of C, X, O, and b waves, as well as Q‐b and Q‐C time intervals, were automatically measured and displayed by means of software in the measurement instrument. Statistical results are listed in Tables 1, 2, 3, and 4.

**Table 1:** C wave amplitudes of AO, PL, PR, LV, and RV. Symbols C_AO_, C_PL_, C_PR_, C_LV_, and C_RV_ denote C wave amplitudes of AO, PL, PR, LV, and RV, respectively. Symbol n is number of subjects in each group. Symbol “M + F” denotes “male and female.” The “30 + 30” denotes 30 males and 30 females. The “90 + 90” denotes 90 males and 90 females

Groups	Sex	*n*	C_AO_ (Ω/s)	C_PL_ (Ω/s)	C_PR_ (Ω/s)	C_LV_ (Ω/s)	C_RV_ (Ω/s)
Younger	M + F	30 + 30	2.118 ± 0.555	1.073 ± 0.277	0.728 ± 0.177	−0.668 ± 0.194	−0.543 ± 0.179
Middle	M + F	30 + 30	1.725 ± 0.560	0.852 ± 0.262	0.609 ± 0.169	−0.487 ± 0.155	−0.408 ± 0.118
Elder	M + F	30 + 30	1.534 ± 0.395	0.761 ± 0.233	0.561 ± 0.180	−0.481 ± 0.171	−0.414 ± 0.167
Total	Male	90	1.615 ± 0.405	0.791 ± 0.225	0.551 ± 0.168	−0.514 ± 0.171	−0.421 ± 0.169
	Female	90	1.969 ± 0.617	0.999 ± 0.308	0.715 ± 0.172	−0.577 ± 0.211	−0.479 ± 0.164
	M + F	90 + 90	1.792 ± 0.557	0.895 ± 0.288	0.633 ± 0.188	−0.545 ± 0.194	−0.455 ± 0.168

**Table 2:** X wave amplitudes of AO, PL, PR, and b wave amplitudes of LV and RV. Symbols X_AO_, X_PL_, and X_PR_ denote X wave amplitudes of AO, PL, and PR, respectively, and symbols b_LV_ and b_RV_ denote b wave amplitudes of LV and RV, respectively

Groups	Sex	*n*	X_AO_ (Ω/s)	X_PL_ (Ω/s)	X_PR_ (Ω/s)	b_LV_ (Ω/s)	b_RV_ (Ω/s)
Younger	M + F	30 + 30	−1.020 ± 0.304	−0.454 ± 0.129	−0.306 ± 0.117	0.288 ± 0.108	0.339 ± 0.120
Middle	M + F	30 + 30	−0.867 ± 0.268	−0.419 ± 0.144	−0.300 ± 0.111	0.224 ± 0.103	0.282 ± 0.129
Elder	M + F	30 + 30	−0.759 ± 0.252	−0.366 ± 0.128	−0.290 ± 0.106	0.219 ± 0.107	0.262 ± 0.104
Total	Male	90	−0.795 ± 0.268	−0.349 ± 0.112	−0.259 ± 0.089	0.219 ± 0.099	0.261 ± 0.094
	Female	90	−0.969 ± 0.296	−0.477 ± 0.132	−0.338 ± 0.117	0.268 ± 0.115	0.327 ± 0.137
	M + F	90 + 90	−0.882 ± 0.294	−0.413 ± 0.138	−0.299 ± 0.111	0.244 ± 0.110	0.294 ± 0.122

**Table 3:** O wave amplitudes of AO, PL, PR, LV, and RV. Symbols O_AO_, O_PL_, O_PR_, O_LV_, and O_RV_ denote the O wave amplitudes of AO, PL, PR, LV, and RV, respectively

Groups	Sex	*n*	O_AO_ (Ω/s)	O_PL_ (Ω/s)	O_PR_ (Ω/s)	O_LV_ (Ω/s)	O_RV_ (Ω/s)
Younger	M + F	30 + 30	−0.049 ± 0.200	0.066 ± 0.117	−0.033 ± 0.081	0.379 ± 0.105	0.224 ± 0.087
Middle	M + F	330	0.002 ± 0.193	0.053 ± 0.123	−0.028 ± 0.079	0.315 ± 0.112	0.189 ± 0.083
Elder	M + F	30 + 30	−0.063 ± 0.176	0.029 ± 0.111	−0.036 ± 0.118	0.267 ± 0.113	0.184 ± 0.082
Total	Male	90	−0.060 ± 0.177	0.035 ± 0.108	−0.039 ± 0.083	0.288 ± 0.107	0.186 ± 0.076
	Female	90	−0.013 ± 0.202	0.062 ± 0.125	−0.026 ± 0.104	0.352 ± 0.122	0.212 ± 0.093
	M + F	90 + 90	−0.036 ± 0.191	0.049 ± 0.119	−0.032 ± 0.094	0.320 ± 0.119	0.199 ± 0.086

**Table 4:** Q‐C intervals of various impedance change components. Symbols Q‐C_AO_, Q‐C_PL_, Q‐C_PR_, Q‐C_LV_, and Q‐C_RV_ denote the Q‐C intervals of AO, PL, PR, LV, and RV, respectively

Groups	Sex	*n*	Q‐C_AO_ (s)	Q‐C_PL_ (s)	Q‐C_PR_ (s)	Q‐C_LV_ (s)	Q‐C_RV_ (s)
Younger	M + F	30 + 30	0.167 ± 0.013	0.160 ± 0.013	0.180 ± 0.028	0.239 ± 0.018	0.255 ± 0.028
Middle	M + F	30 + 30	0.174 ± 0.017	0.163 ± 0.017	0.171 ± 0.032	0.243 ± 0.021	0.258 ± 0.026
Elder	M + F	30 + 30	0.177 ± 0.019	0.166 ± 0.016	0.168 ± 0.029	0.247 ± 0.020	0.256 ± 0.030
Total	Male	90	0.178 ± 0.017	0.168 ± 0.016	0.181 ± 0.032	0.246 ± 0.019	0.257 ± 0.027
	Female	90	0.168 ± 0.016	0.158 ± 0.014	0.164 ± 0.025	0.240 ± 0.020	0.255 ± 0.029
	M + F	90 + 90	0.173 ± 0.017	0.163 ± 0.016	0.173 ± 0.030	0.243 ± 0.020	0.256 ± 0.028

Note

Q‐b intervals of LV and RV for 180 normal adults are 0.126 ± 0.022 s and 0.126 ± 0.021 s, respectively.

### 2.2 Participants

The participants were 180 normal healthy adults. Their ages ranged from 18 to 78 years, and their average age was 50.4 ± 15.4 years. The systolic pressure of the participants was between 95 to 138 mmHg, and diastolic pressure was between 65 and 89 mmHg. All subjects were asked about their medical history, and their ECG, blood pressure, heart rate, and physical examination were all normal. The participants were divided into three age groups according to WTO rules: younger (18–44 year), middle (45–59 year), and elder (60–78 year). 30 males and 30 females were included in each age group.

### 2.3 Statistical analysis

Statistical analysis was performed by means of SPSS12.0 software. The measurement results of C, X, O, b wave amplitudes, as well as the Q‐b and Q‐C time intervals, were all expressed with the average value and standard deviation. The difference level between two averages was judged by *t* test. If *p‐*value < .05, there was a significant difference between two averages.

## 3 RESULTS

The measurement results of C, X, O, and b wave amplitudes, as well as Q‐C intervals of differential graphs of RICG, are listed in Tables 1, 2, 3, and 4, respectively. Q‐b intervals of LV and RV can be found in the footnote of Table 4. It can be seen from the measured data in the four tables and waveform graphs in Figure 4 that:

1. The waveform of PL, PR, and AO was upward in systolic phase, but that of LV and RV was downward. This shows that the waveforms of both were in opposite phases.
2. The waveform amplitudes of C and X waves of various components, as well as that of b and O waves of LV and RV, all decreased as age increased (Tables 1, 2, and 3), and there were significant differences between the younger and elder groups (*p* < .05), except for X wave of PR (*p* > .40). The waveform amplitudes of the female group were larger than those of the male group, *p* < .05.
3. Among the five components, the C wave amplitude of AO was largest, that of PL and PR was next, while that of LV and RV was smallest (See Table 1). The O wave amplitudes of LV and RV were larger than those of AO, PL, and PR, *p* < .01 (Table 3).
4. The Q‐C intervals of AO, PL, and LV increased as age increased (Table 4). The Q‐C intervals of AO, PL, and PR of the male group were bigger than those of the female group, *p* < .05. The Q‐C intervals of LV and RV were longer than those of AO, PL, and PR, *p* < .01 (See Table 4). The Q‐b intervals of LV and RV were smaller than the Q‐C intervals of AO, PL, and PR (Table 4 footnote).

## 4 DISCUSSION

### 4.1 Impedance change component AO

As shown in Figure 4, the C wave of the differential graph of AO appears after ECG QRS waves, and it is a high positive wave. Its starting point corresponds to the first cardiac sound (S1). The semilunar valves of the aorta open at this time, and the ejection of left ventricle starts. The peak point is reached after 0.05–0.07 s, and subsequently, it decreases until it reaches baseline levels. The C wave of AO is a result of rapid ejection of the left ventricle and rapid expansion of the aortic volume in systolic phase. This reflects indirectly the speed change of aortal expansion. The average C wave amplitude of normal subjects was 1.792 ± 0.557 Ω/s (see Table 1).

C wave amplitude of AO is related to the contractile force and stroke volume of the left ventricle. If C wave amplitude is larger, it suggests that the myocardial contractility of the subject is stronger and that stroke volume is larger. Thus, C wave amplitude of AO is an important parameter reflecting the contractile function of the left ventricle. The contractile function of left ventricle generally decreases as age increases. The C wave amplitude of AO also decreases as age increases (see Table1), and there is a significant difference between C wave amplitudes of the younger and elder groups (*p* < .001).

In addition, the C wave amplitudes of 196 patients were also measured. The C wave amplitude of 36 patients with coronary heart disease (first group) was 1.145 ± 0.360 Ω/s, that of 35 patients with hypertension (second group) is 1.216 ± 0.441 Ω/s, that of 72 patients with cardiac insufficiency (third group) is 0.760 ± 0.300 Ω/s, and that of 53 patients with hyperthyroidism (fourth group) is 3.029 ± 1.139 Ω/s. These data show that the contractile function of patients in the first, second, and third groups is remarkably decreased, while that of the fourth group is abnormally strengthened. Thus, C wave amplitude of AO can reflect the pathological changes of patients.

The X wave of AO is a negative wave after the C wave and is in the later stage of left ventricle contraction. Its negative peak point X is in the vicinity of S_2_. The X wave is caused by the recovery of aortal volume, and it reflects indirectly the speed change of aortal recovery. The mean of X wave amplitude in 180 normal adults was −0.882 ± 0.294 Ω/s (see Table 2). The O wave of AO is a positive or negative wave in its initial diastolic stage, and its amplitude is smaller. The O wave mean of 180 normal adults is −0.036 ± 0.191 Ω/s (see Table 3).

### 4.2 Impedance change components PL and PR

Figure 4 shows that there is also a positive C wave in differential graphs of PL and PR, and it is in time phase of the C wave of AO. The C waves of PL and PR are a result of rapid ejection of blood form the right ventricle and volume expansion of the pulmonary arteries in systolic phase. It reflects indirectly the speed change of volume expansion of pulmonary arteries. The average C wave amplitudes of PL and PR are 0.895 ± 0.288 Ω/s and 0.633 ± 0.188 Ω/s, respectively (see Table1). The amplitude of PL is significantly bigger than that of PR (*p* < .001).

There is also a negative X wave in PL and PR, and it is approximately in time phase of the X wave of AO. The X waves of PL and PR are a result of volume recovery of the pulmonary arteries and reflect indirectly the speed change of volume recovery of pulmonary arteries. The average X wave amplitudes of PL and PR are 0.413 ± 0.138 Ω/s and 0.299 ± 0.111 Ω/s, respectively (see Table 2). The amplitude of PL was significantly larger than that of PR (*p* < .001).

The positive or negative O wave appears in PL and PR after the X wave. The average O wave amplitudes of PL and PR are 0.049 ± 0.119 Ω/s and −0.032 ± 0.094 Ω/s, respectively (see Table 3). O wave amplitude of PL is bigger than that of PR, *p* < .001. The ratio O/C of O wave average to C wave average for PL and PR is usually smaller for healthy individuals, and the mean of O/C was 0.011 ± 0.138. Large O waves and small C waves appear often in PL and PR for patients with cardiac insufficiency; thus, the ratio O/C in these patients is larger (Kuang et al., 2014).

### 4.3 Impedance change components LV and RV

As shown in Figure 4, there are b, C, and O waves in LV and RV. The b wave appears in initial systolic stage, and it is a small positive wave. The b wave amplitudes of 180 normal adults are 0.244 ± 0.110 Ω/s and 0.294 ± 0.122 Ω/s for LV and RV, respectively (see Table 2), and b wave amplitude of RV is bigger than that of LV, *p* < .001. Q‐b interval of LV and RV is shorter than Q‐C interval of AO, PL, and PR (*p* < .001), because b wave peak point of LV and RV is in front of C wave peak point of AO, PL, and PR in time phase. On the basis of physiological principles and resistance law, if the ventricle volume diminishes, its impedance should increase as the ventricle ejects the blood. However, the positive b wave in Figure 4 shows that the impedance of the ventricle diminishes in initial systolic stage. It is likely that shortening of the ventricle length is earlier than the contraction of ventricular sectional area in the initial systolic stage (Brecker & Stephen, 2000), and the impedance of ventricle is reduced as a result.

After the b wave, a negative C wave appears in LV and RV, which is the main wave of LV and RV. These C waves are caused by rapid contraction of the ventricles. Since the C wave peak points of LV and RV are after the C wave point of AO, PL, and PR, the Q‐C intervals of LV and RV are longer than that of AO, PL, and PR (see Table 4), *p* < .001. C wave amplitudes of LV and RV are −0.545 ± 0.194 Ω/s and −0.455 ± 0.168 Ω/s in 180 normal adults, respectively (see Table 1). The amplitude of LV is significantly larger than that of RV, *p* < .001.

The O wave of RV and LV appears after S_2_ and is caused due to rapid engorgement of the ventricles in the initial diastolic stage. O wave amplitudes of LV and RV are 0.320 ± 0.119 Ω/s and 0.199 ± 0.086 Ω/s in 180 normal adults, respectively (see Table 3), and the amplitude of LV is significantly larger than that of RV (*p* < .001). The O wave amplitudes in RV and LV decrease as the age increases, and there is a significant difference between the measured data of the younger and elder groups (*p* < .01). O wave amplitudes of RV and LV for patients with cardiac insufficiency are remarkably decreased. For instance, the O wave amplitudes of RV and LV for 72 patients with cardiac insufficiency are −0.130 ± 0.090 Ω/s and −0.109 ± 0.105 Ω/s, respectively, and they are smaller than that of the adults with normal function (see Table. 3, *p* < .001).

### 4.4 Analysis of measurement results

Figure 3 shows the waveform graphs of impedance change components of RICG. The main waveforms of the vascular components AO, PL, and PR are upward in systolic phase, while those of the ventricular components LV and RV are downward, and they are in opposite phase. This corresponds with the fact that the ventricular volume diminishes while the volume of blood vessels expands in the systolic phase (Da‐Nian, 2009). It is known from physiology that when the semilunar valves of the aorta begin to close, S_2_ occurs and the ventricular volume is minimal. In Figure 3, the negative peak points of LV and RV are in phase with S_2_. This means that the time phase of the negative peak point of the impedance change components of LV and RV corresponds with that of minimal ventricular volume (Da‐Nian, 2009). It is evident that the waveform graphs of various impedance change components of RICG obtained through waveform separation can reflect indirectly the physiological activity of the heart and blood vessels in thorax.

It could be proven theoretically that the impedance change on chest surface caused by blood vessels is larger when the included angle between the blood vessels and human body axis (i.e., electrical current line) is smaller (Qiu‐Jin et al., 2012). Conversely, the impedance change caused on the chest surface is smaller when the included angle is larger. This is precisely a parallel effect in the measurement of the electrical impedance graph. It can be known from physiology that the blood volume discharged by the left ventricle is in concordance with that of the right ventricle. The measurement results show that the C wave amplitude (1.792 ± 0.557 Ω/s) of AO was significantly bigger than the sum (1.527 ± 0.382 Ω/s) of C wave amplitudes of PL and PR. This difference is related to the strike of blood vessels in thorax.

Anatomically, the aorta in the thorax is basically parallel with the human body axis. Thus, it can be expected that the impedance change that the aorta induces on the chest surface would be larger. But the distribution of the large blood vessels in the lung is more complex. The included angle between a part of pulmonary arteries and the human body axis is larger, and these blood vessels induce only smaller impedance change on chest surface. The included angle between other part of pulmonary arteries and human body axis is smaller, and they can induce bigger impedance changes on the chest surface. It is known from the anatomy that the volume of the right lung is bigger than that of the left lung, and the blood volume of the right lung is larger than that of the left lung. Accordingly, the C wave amplitude of PR should be bigger than that of PL, but the results in Table1 show that C wave amplitude (0.633 ± 0.188 Ω/s) of PR was smaller than that (0.895 ± 0.288 Ω/s) of PL. This may be caused by the fact that the included angle between the right pulmonary arteries and the human body axis is larger.

It is known from physiology that the volume change amplitude of the ventricle is equal to stroke volume (Da‐Nian, 2009), and it should be bigger than the volume change of the aorta between the detection electrodes (E, E'). However, the C wave amplitudes of LV and RV were only approximately 25%–33% of C wave amplitude in differential graph of AO (Table1). This indicates that the large volume change of the ventricles evokes only a small impedance change on the chest surface. It is well known that the major and minor axes of the ventricles shorten simultaneously when the heart contracts. On the basis of resistance law, the shortening of major axes reduces the ventricle impedance, and the shortening of minor axes leads to an increase of ventricular impedance. These two opposite impedance changes counteract each other. Thus, the impedance change that the ventricle causes on chest surface is smaller.

In order to elucidate the origin of thoracic impedance change, many scholars made the devil of experiments. Two representative experimental results are valid: First is the dog experiment performed by Tonmuson FD (Ju‐kang & Kai‐bai, 1992). The results of this study shown that the descending percentage of C wave amplitude of ICG's differential graphs was 48% when occluding the aorta, and 46% when occluding the pulmonary artery. Thus, the contributions of the aorta and pulmonary arteries are basically in concordance. Second is the artificial heart experiment performed by Baker LE and Mistry GD (Baker & Mistry, 1981). The results of this study have shown that the left and right heart circulation contributes approximately 60% and 40% to thoracic impedance change, respectively. The data in Table1 show that C wave amplitudes of AO, PL, and PR are 1.792 ± 0.557 Ω/s, 0.895 ± 0.288 Ω/s, 0.633 ± 0.188 Ω/s, respectively. By these data, the contributions of AO and PL + PR to the thoracic mixed impedance change can be proximately computed, respectively. The contribution of AO is 54.0%, and that of PL + PR is 46.0%. These results are in approximate agreement with those in the two experiments mentioned above.

In order to obtain the impedance change components that reflect the physiological activity of the heart and large blood vessels in thorax, many interventions were performed in our study. In the measurement circuit, the isolated direct current source was induced to ensure the safe measurement of human body, and the synchronization detection circuit was applied to elevate the property of opposing the interference. The five impedance change components of RICG were obtained through establishing and solving thoracic impedance equations. Model error and measured error were included in these equations; thus, the thoracic impedance equations were the contradict algebraic equations (Gong‐Yan, 2000). If the solutions are computed with usual method, the waveform graphs, which correspond to physiological changes, cannot be obtained. For this reason, the algebraic reconstruction technique, already used in the first X‐CT system, was adopted to calculate the solution of the thoracic impedance equations (Gong‐Yan, 2000).

Our research has evolved the clinical applications of RICG (for instance, formula of measuring the cardiac output, ejection fraction, and myocardial oxygen consumption), in which, the square root formula for measuring the cardiac output had been published in Med. Phys (Kuang et al., 2018).

## 5 CONCLUSION

The differential graphs of each impedance change component of RICG for 180 normal adults were measured in this paper, and statistical analysis was performed according to the different age and sex groups. C and X wave amplitudes of various components all decrease as age increases. The wave amplitudes of the male group were smaller than those of the female group, while the Q‐C interval of the male group was longer than that of the female group. C wave amplitude of AO was largest, that of PL and PR was next, and that of LV and RV was smallest. The differential waveform graphs of various impedance change components of RICG may reflect indirectly the physiological activity and pathological changes of the heart and of the large blood vessels in thorax.

## References

[anec12714-bib-0001] Baker, L. E. , & Mistry, G. D. (1981). Assessment of cardiac function by electrical impedance. Proceeding of 5th ICEBI (pp. 7–10). Tokyo, Japan.

[anec12714-bib-0002] Brecker, S. J. , & Stephen, J. D. (2000). The importance of long axis ventricular function. Heart, 84(6), 577–579. 10.1136/heart.84.6.577 11083726 PMC1729502

[anec12714-bib-0003] D'Ambrosio, A. , Cotoia, A. , Beck, R. , Salatto, P. , Zibar, L. , & Cinnella, G. (2018). Impedance cardiography as tool for continuous hemodynamic monitoring during cesarean section: Randomized, prospective double blind study. BMC Anesthesiology, 18, 32–40. 10.1186/s12871-018-0498-4 29587655 PMC5870261

[anec12714-bib-0004] Da‐Nian, Z. (2009). Physiology (7th ed., pp. 79–81). Beijing, China: People health publishing company.

[anec12714-bib-0005] DeMarzo, A. P. (2013). Using impedance cardiography to detect asymptomatic cardiovascular disease in prehypertensive adults with risk factors. High Blood Pressure and Cardiovascular Prevention, 20(2), 61–67. 10.1007/s40292-013-0009-0 23609893

[anec12714-bib-0006] Ebrahim, M. , Hegde, S. , Printz, B. , Abcede, M. , Proudfoot, J. A. , & Davis, C. (2016). Evaluation of impedance cardiography for measurement of stroke volume in congenital heart disease. Pediatric Cardiology, 37, 1453–1457. 10.1007/s00246-016-1456-x 27562130

[anec12714-bib-0007] Gong‐Yan, L. (2000). Teaching materials of the mathematical model (pp. 88–91). Beijing, China: Publishing Company in Beijing University.

[anec12714-bib-0008] Heinink, T. P. , Lund, J. N. , & Williams, J. P. (2015). Accuracy of impedance cardiography for evaluating trends in cardiac output. British Journal of Anaesthesia, 115(2), 322–323. 26170358 10.1093/bja/aev243

[anec12714-bib-0009] Ju‐kang, G. U. , & Kai‐bai, D. (1992). Clinical cardiac function (pp. 186–187). Hefei, China: Anhui Science and Technique Publishing Company.

[anec12714-bib-0010] Kuang, M. X. , Kuang, S. J. , Qiao, Q. J. , Kuang, N. Z. , Zhao, H. , & Cheng, X. L. (2018). Comparing square root method of measuring the cardiac output by means of aortic impedance change component to Kubicek's method. Medical Physics, 45(9), 4297–4305. 10.1002/mp.13076 29963701

[anec12714-bib-0011] Kuang, M. X. , Xiao, Q. J. , Cui, C. Y. , Kuang, N. Z. , Hong, W. Q. , & Ai‐Rong, H. U. (2010). Mechanism of the formation for thoracic impedance change. Annals of Biomedical Engineering, 38(3), 1007–1016. 20336823 10.1007/s10439-009-9886-8

[anec12714-bib-0012] Kuang, N. Z. , Xiao, Q. J. , He, B. Q. , Jing‐Juon, F. U. , & Kuang, M. X. (2014). Analyzing the formation of normal and abnormal O waves in thoracic impedance graph using the impedance change components for aorta, blood vessels in lung and ventricles. Cardiology Journal, 21(2), 176–182. 10.5603/CJ.a2014.0014 24526509

[anec12714-bib-0013] Kubicek, W. G. , Karnegis, J. N. , Patterson, R. P. , Witeose, D. A. , & Mattson, R. H. (1966). Development and evaluation of impedance cardiac output system. Aerospace Medicine, 37(12), 1208–1212. 5339656

[anec12714-bib-0014] Ming‐Xing, K. , Qiu‐Jin, X. , Nan‐Zhen, K. , Chao‐Ying, C. , & Ai‐Rong, H. U. (2011). Studies on separating the impedance change components of blood vessels and ventricles in thorax from mixed impedance signals on chest surface. Medical Physics, 38(6), 3270–3278. 21815401 10.1118/1.3594548

[anec12714-bib-0015] Patterson, P. R. (1985). Sources of the thoracic cardiogenic electrical impedance signal as determined by a model. Medical and Biological Engineering and Computing, 23, 411–417. 10.1007/BF02448927 4068776

[anec12714-bib-0016] Qiu‐Jin, X. , Zhen, W. , Ming‐Xing, K. , Ping, W. , Pei, L. , & Jian‐Feng, J. I. (2012). Thoracic impedance change equation deduced on the basis of parallel impedance model and Ohm's law. Medical Physics, 39(2), 1042–1045. 10.1118/1.3678987 22320814

[anec12714-bib-0017] Rada, M. A. , Cuffaro, P. E. , & Galarza, C. R. (2014). Predictive value of non‐invasive hemodynamic measurement by means of impedance cardiography in hypertensive subjects older than 50 years of age. Clinical and Experimental Hypertension, 36(5), 280–284. 10.3109/10641963.2013.810232 24047376

[anec12714-bib-0018] Wang, L. , & Patterson, R. (1995). Multiple sources of the impedance cardiogram based 3‐D finite difference human thorax models. IEEE Transactions on Biomedical Engineering, 42, 141–149. 7868141 10.1109/10.341826

[anec12714-bib-0019] Wang, T. , Zhang, Y. , Li, Q. X. , Li, Q. X. , Jia, S. M. , Shi, C. J. , … Liu, B. (2017). Acute kidney injury in cancer patients and impedance cardiography‐assisted renal replacement therapy: Experience from the onconephrology unit of a Chinese tertiary hospital. Experimental and Therapeutic Medicine, 14, 5671–5677. 10.3892/etm.2017.5244 29285109 PMC5740768

